# Supplementary figures and images for: Anticipation-induced delta phase reset improves human olfactory perception
Source: PLoS Biol. 2020 May 26;18(5):e3000724. doi: 10.1371/journal.pbio.3000724 (PMC7250403; doi:10.1371/journal.pbio.3000724)

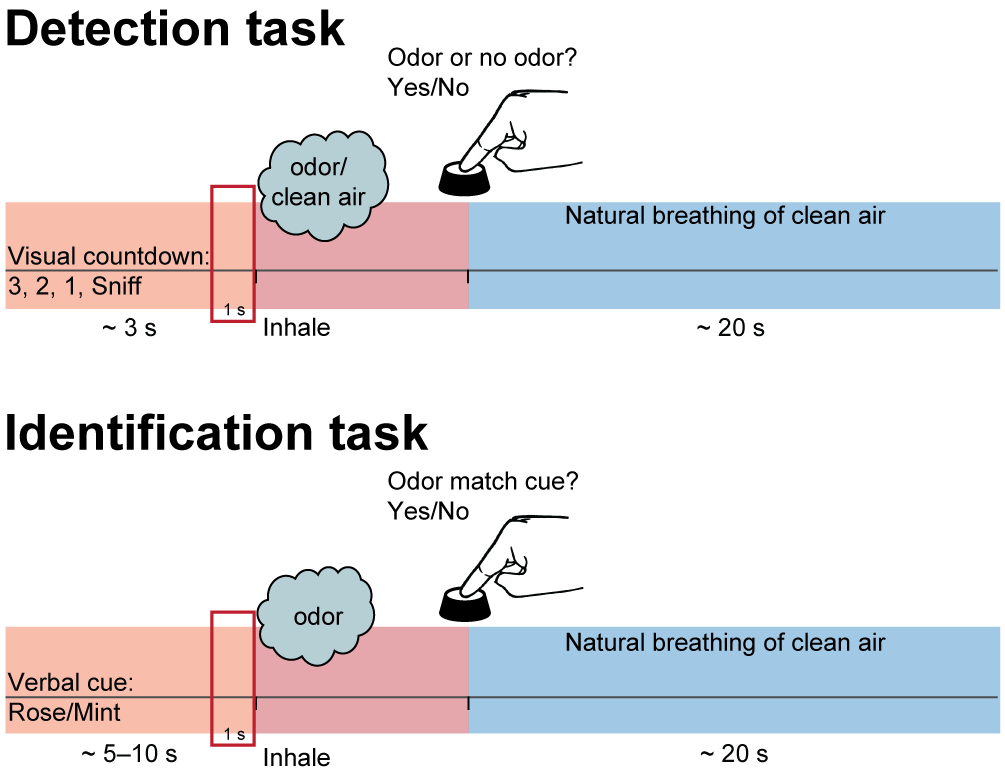

Supplement: S1 Fig — Related to Fig 1. Following selection of data sets according to our inclusion criteria (described in detail in the main text), data from two olfactory tasks were included. In a detection task (top panel), the participants were presented with either odorized or odorless air following a visual cue and indicated whether an odor was present via button press. In an identification task (bottom panel), the participants were presented with an odor following an auditory cue and indicated whether the odor matched the cue via button press. The red outlined area (box just prior to inhale onset) indicates the time window of interest in our analyses, specifically, the pre-inhale anticipatory period. (TIF) [file pbio.3000724.s001.tif]
