# Supplementary material for: Anticipation-induced delta phase reset improves human olfactory perception
Source: PLoS Biol. 2020 May 26;18(5):e3000724. doi: 10.1371/journal.pbio.3000724 (PMC7250403; doi:10.1371/journal.pbio.3000724)
Supplement: Attachment — Submitted filename: ResponseToReviewers.docx [file pbio.3000724.s002.docx]

Dear Editor,

Thank you for this constructive peer review of our work. We have addressed every comment raised by the reviewers, and our point-by-point responses are below, in blue font. In response to the reviewer’s comments, we have conducted additional analyses, including re-running time frequency analyses with a shorter filter window length and computing broad band averages. We also modified Fig 4 to include these results.

**Reviewer #1**: This is an outstanding and well written paper describing possible neural mechanisms underlying anticipation's effect on odor perception -- namely the well established influence of anticipation/attention on odor perception. The results are remarkably well presented, methods carefully described and the introduction and discussion well crafted with a crisp scholarly tone. While this manuscript is human iEEG, the authors do service to this study by placing their results in context with those from the non-human literature. Compelling iEEG of this sorts is exceptionally rare in the olfactory system and thus this project has a high level of innovation and the outcomes, that delta phase resetting independent of sensori-motor influences, may sculpt perception, is significant. This work will be of impact to the olfactory community but more than that, to the wealth of researchers exploring the brain basis for attention.

We have no major issues, nor any true minor issues for that matter -- clearly this manuscript has been polished by the authors over rounds of careful consideration.

Thank you for your positive comments on our work. We are very excited to hear that you feel our work is impactful and innovative.

Small items for correction:

1)Line 483: What exactly is meant by power modulations of higher Hz (perhaps cite a fig here to guide readers)?

Thank you for noticing this error on our part. This sentence is describing an analysis that we ran, but ended up leaving out of the final version of the manuscript, in order to keep the manuscript focused. We have removed this sentence from the manuscript.

2)Fig 1: one of the pink shaded boxes overshadows the raw LFP trace.

Thank you for bringing this to our attention. We have fixed the figure.

3)The last paragraph in the Discussion on neuroD disorders, etc, is super tangential. We get it, but, wouldn't this paper end more compelling with highlighting really why IT is great (as well covered in the previous paragraphs) versus needing to end with a weak translational spin-off/foreshadow?

We agree with you, and have removed it from the manuscript.

**Reviewer #2**: The study by Ghazaleh et al. describes how the phase of low frequency local field potential (LFP) oscillations recorded by intracranial EEG in humans are modulated in the piriform cortex by the anticipation of an odor prior to sniffing. The study is important, as most research thus far targeting the mechanisms of attention was focused on the auditory and visual systems, which have markedly different anatomy. Therefore, it is not clear if the same principles can be applied to the olfactory system. Ghazaleh et al. provide convincing evidence that phase reset, which is a major mechanism of attention in other sensory systems, is also engaged by olfactory perceptual processes. I reviewed a previous version of the manuscript for a different journal, and I find this version much improved. In fact, I only have a couple of minor criticisms/suggestions.

We are very happy to hear that you feel the manuscript is much improved, and are thankful for your encouraging comments (and for your previous review of our manuscript, which was extremely helpful and led to an improved manuscript).

1)      I have a hunch that the "odor-evoked theta power increase" in the olfactory anticipation condition might be explained by the consistent delta phase reset and phase amplitude coupling. Even if the authors disagree, this possibility should be mentioned and disputed.

We agree. This is an interesting possibility, and could be true. We now raise this point in the manuscript on lines 354-358.

2)      In the results, the frequency range 0-2 Hz or 0-8 Hz does not really make sense, as there were no measurements made at 0 Hz. Therefore, I suggest changing it to 0.5 - xxx Hz everywhere, as based on the methods, 0.5 Hz was the low end of the frequency spectrum tested.

Excellent point. We have made this change throughout the manuscript.

**Reviewer #3, Nathan Weisz**: This is an interesting paper investigating time-resolved neuronal dynamics in olfactory (piriform) cortical strutures, during a pre-inhalation period when human subjects either anticipated or did not anticipate an odour presentation. Measurements were done in the context of presurgical epilepsy diagnosis using sEEG directly from relevant cortical structures. The authors show in particular increased intertrial phase locking in a delta frequency range prior to inhalation when individuals were anticipating an odour. This result was correlated with post-inhalation power modulations as well as behavioural performance. The authors show that the effect is not correlated with post-stimulus power changes as well as overt inhalation responses. Overall, the study points to similar anticipation / attention (not dissociable given the current design) related phase adjustment in a slow frequency range, similar as it has been shown in other sensory modalities.

Given the unique architecture of the olfactory system, this is an interesting study that is a strong candidate for Plos Biology.

Thank you for your positive comments, we are happy to hear that you find our study interesting.

For me the most critical issue is whether the main effect is indeed a unique anticipation related pre-inhalation effect. I do not find this completely conclusive given the presentation of the results. In particular it can be seen in Figure 2A that the peak (at ~.7 Hz) is clearly post-stimulus, without an obvious distinct prestimulus peak. Depending on the settings of the time-frequency analysis, the pre-inhalation effects (in particular at this low frequency) could be a "bleeding effect" of a post-inhalation peak into the prestimulus period. I would suggest rerunning the time-frequency analysis using an FFT on hanning-tapered windows of varying lengths. If the pre-inhalation effect shrinks in the post-inhalation direction, then I would be worried about the current interpretation.

Thank you for your suggestion. We agree it is important to consider the possibility that filter smearing impacted our findings. In response to your concern, we have re-analyzed our data using a shorter window length (2cpf) and you suggested, and include the result in a new version of Fig 4. We believe that these changes have led to a more robust set of findings.

We carefully controlled for any potential power modulations present due to filter smearing that could have impacted our phase estimates across conditions (see Fig 4, which includes a systematic analysis of any power differences across conditions, and any correlations between power modulations and differences in ITC across conditions). However, we did not consider the possibility that filter smearing might have impacted our phase estimates as well. Therefore in response to your suggestion, we followed the method outlined in a study that performed a similar control (*Andreas Wutz, Nathan Weisz, Christoph Braun and David Melcher: Temporal windows in visual processing: Prestimulus brain state…; Journal of Neuroscience, 2014*). In this study, a second, smaller window length was used corresponding to 2 cpf, and similar results were found. Excerpt from the methods of this paper: “we confirmed that the reported findings are not dependent on the length of the windowing function, since similar effects were found using a shorter window length (2 cpf; data not shown)”.

In line with these published methods, we therefore re-ran our time-frequency analyses using a window corresponding to 2 cps. This control analysis yielded virtually identical results, with statistically significant ITPC evident beginning right at 500ms prior to odor onset, strongly suggesting that our results are not merely due to filter smearing effects. We now mention this control analysis in the manuscript on lines 304-307, and include the result in a new version of Fig 4.

Furthermore, it would be also a good idea to present the broadband evoked responses: is a low frequency difference visible before spectral analysis? is there a post-stimulus difference in terms of latencies (e.g. an earlier peak when anticipating an odour).

We appreciate than an analysis of ERPs during anticipation of odor could yield additional interesting findings. However, as the focus of our study is on oscillations and specifically, phase dynamics, we feel that an ERP analysis is beyond the scope of our study, and including it would risk diluting the main message. That said, in response to this concern, we computed average broad-band (raw) epochs for each subject, baseline-corrected to the 1s time window prior to inhale onset, to look for a post-stimulus difference in terms of latencies. We found no statistically significant differences across conditions. We feel this will be an interesting topic for a future study, and deserves a more thorough and careful consideration, but such analyses would require at least several more months of data analysis in order for us to generate any definitive conclusions.

The authors report an absence of power differences. This by itself is not shocking, however the spectra that are presented in Figure 4 look very "1/f"-ish without any clear peaks. Wouldn't this be expected when recording from medial temporal lobe structures (e.g. a theta peak).

This is an interesting point, thank you for bringing it to our attention. We agree that a theta peak would typically be visible in some medial temporal lobe structures like the hippocampus. However, we were recording from primary olfactory cortex, specifically piriform cortex, and the spectral properties of this region are not well understood, and have not been fully characterized in humans. Therefore, we remain agnostic as to whether a clear peak in a particular frequency range should be measurable in this brain region. We now mention this point in the manuscript on 278-281.

Also, it would be a good idea to display the t-map in 4B using a more meaningful minimum / maximum (the color bar says -30 to +30).

Thank you for your suggestion. Upon further reflection, we decided to remove the t-map in order to make space to add a panel showing the new control analysis for filter smearing. We believe that since we found no significant differences in pre-inhale power, showing the t-map does not add much to the figure, and would only be showing random, non-significant, small power modulations that carry no meaning.

In their discussion the authors mention fMRI studies showing anticipation related differences (e.g. p. 22 ll. 441). In the present study effects are only found in terms of intertrial phase coherence. How can these effects be reconciled?

This is a very relevant question. We would speculate that because fMRI is not a direct measure of neural activity, but rather a measure of blood flow, there are a number of possible neural modulations that might impact fMRI signals. For example, it is possible that the occurrence of a phase shift in the brain might be measurable indirectly using fMRI. We now include mention of this in the manuscript on lines 515-516.

Minor:

-p. 5, l. 97:  "enhances olfactory response ..." --> "improves"?

We have made the requested change.

-In my personal opinion the long excursion into Predictive Coding on p. 23 can be removed without much loss in substance.

Thank you for the suggestion. Though we partially agree, we have decided to keep the paragraph because we feel our findings do have relevance to predictive coding models, and we would like to cite these studies in our manuscript. We modified the paragraph in an attempt to make it more relevant.
